# Clinical Use of Extracellular Vesicles in the Management of Male and Female Pattern Hair Loss: A Preliminary Retrospective Institutional Review Board Safety and Efficacy Study

**DOI:** 10.1093/asjof/ojac045

**Published:** 2022-05-24

**Authors:** Gordon H Sasaki

**Affiliations:** plastic surgeon in private practice in Loma Linda, CA, USA

## Abstract

**Background:**

Pattern hair loss is a common disorder in female and male patients.

**Objectives:**

To assess the safety, efficacy, and satisfaction of a single extracellular vesicle (EV) treatment over 6 months.

**Methods:**

A retrospective open-label study among 22 female and 9 male patients who demonstrated early stages of alopecia or were in remission from previous medical and surgical treatments. The amount of undiluted or diluted volumes of EV solution used was determined by the extent and degree of alopecia. Global photography, Patient Global Aesthetic Improvement Scale (PGAIS) and Investigator Global Aesthetic Improvement Scale (IGAIS) questionnaires, and trichoscan measurements were compared at baseline and 6 months in 3 response categories.

**Results:**

Frequent growth responses were observed: older aged females and younger aged males, shorter history of alopecia, earlier stages of hair loss, larger and undiluted volumes of XoFlo, previous positive responses to medical and surgical treatments, and absence or control of disease factors affecting the hair. Global photography, trichoscan for density, follicle diameter, terminal: vellus ratio, and PGAIS/IGAIS satisfaction questionnaires at baseline and 6 months were useful in assessing clinical efficacy. No significant adverse reactions were observed.

**Conclusions:**

Intradermal injections with varying doses of EVs were safe and effective among indicated alopecic female and male patients. Findings suggest that the presence of positive factors, absence of conditions known to negatively affect hair growth, and administration of larger volumes of XoFlo may have a significant influence on the use of this new cell-free therapy.

**Level of Evidence: 4:**

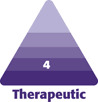

Male and female pattern loss of hair is a prevalent disorder often with early onset that can lead to significant psychosocial effects in both sexes.^1-4^ Regardless of the etiological factors in play, the pathological changes appear nearly identical in males and females and include, but not limited to, shortened anagen phases and progressive follicular miniaturization in subsequent catagen/telogen stages. With the burden of genetic transmission and the presence of environmental stresses, these latent follicular remnants may further advance to exogen shedding and kenogen deletion, leading to an irretrievable reduction of thickness, density, and total number of hairs.^5^ Although male pattern hair loss (MPHL) is believed to be due to a polygenic mode of inheritance and presence of dihydrotestosterone (DHT), the precise biomolecular mechanisms are unclear.^6^ Female pattern hair loss (FPHL) has remained a poorly understood complex in which a number of factors, such as heredity, androgen-dependency and other hormonal effects, inflammation, immunological diseases, drugs, acute stress, diet, significant weight changes, and partum, continue to be investigated.^7-10^ Follicular unit extraction surgery^11-14^ remains the gold standard treatment for advanced MPHL and FPHL as a stand-alone therapy because viable anatomically intact follicles are transplanted from genetically protected sites to alopecic areas. In contrast, medical treatments are believed to promote neogenesis or rescue of dormant hair follicles in susceptible areas of the scalp whose eventual fate might be one of thinning and loss. FDA-approved drugs, such as finasteride, which modulate androgen metabolism, or minoxidil that promote hair growth, have limited success rates, suggesting that additional pathogenic pathways may be at play that require poly-therapies and prolonged treatments for lasting results.^15^

For over 20 years, culture and animal follicle studies^16,17^ have demonstrated that paracrine communicators, emanating from stem cell domains, not only play critical roles in defining cell identity, self-renewal, differentiation, and migration during de novo follicle morphogenesis but also regulate recapitulation of its embryonic development during postnatal growth, miniaturization, and restoration. Knowledge of these cellular and molecular regulators^18,19^ has provided insights for the development of innovative stem cell clinical trials and therapies in the field of regenerative medicine to address human aesthetic and diseased hair restoration. Current investigations and clinical protocols have focused enthusiastically on the use of adipose-derived stem cells (ADSCs),^20-23^ platelet particles in platelet-rich plasma (PRP),^24-32^ and isolated biologic products in conditioned media^33-35^ to promote neogenesis of new follicles, stimulate the transition from telogen to anagen, and recover apoptotic follicles. The putative mechanisms of action are directly related to the myriad of growth factors, cytokines, and other active cellular biological molecules that orchestrate hierarchical signaling and transcriptional cross-talk from domains in the dermal papilla to proximal follicular stem cell niches (bulge) and their corresponding receptors downstream in the secondary hair germ and matrix. Ultimately gene-expression factors promote hair growth and regeneration of preferred cell populations within normal postnatal or miniaturized follicles.

Extracellular vesicles (EVs) are being investigated through clinical trials and studies because their diverse released bioactive molecules are vital for intercellular communications and display similar paracrine effects as growth factors and cytokines discharged from mesenchymal stem cells (MSCs) and PRP (Figures 1, 2). The responses include cellular proliferation and differentiation, angiogenesis, immuno-modulation, and regulation of inflammation all of which have potential therapeutic uses in cardiovascular diseases,^36^ muscle-cartilage-bone diseases,^37,38^ wound healing,^39^ hair loss,^40-44^ and other diseases.^45,46^ Although hair restoration surgeons are citing impressive and safe results with EV treatments for hair restoration, no evidence-based data have been published.^47,48^ The use of EVs biologicals, as a cell-free therapy, has recognized advantages over cell-based therapies that include allogenic usage, superior safety profile, less immunogenicity, “off-the-shelf” strategy, stability, and scalability.^49-51^

**Figure 1.**
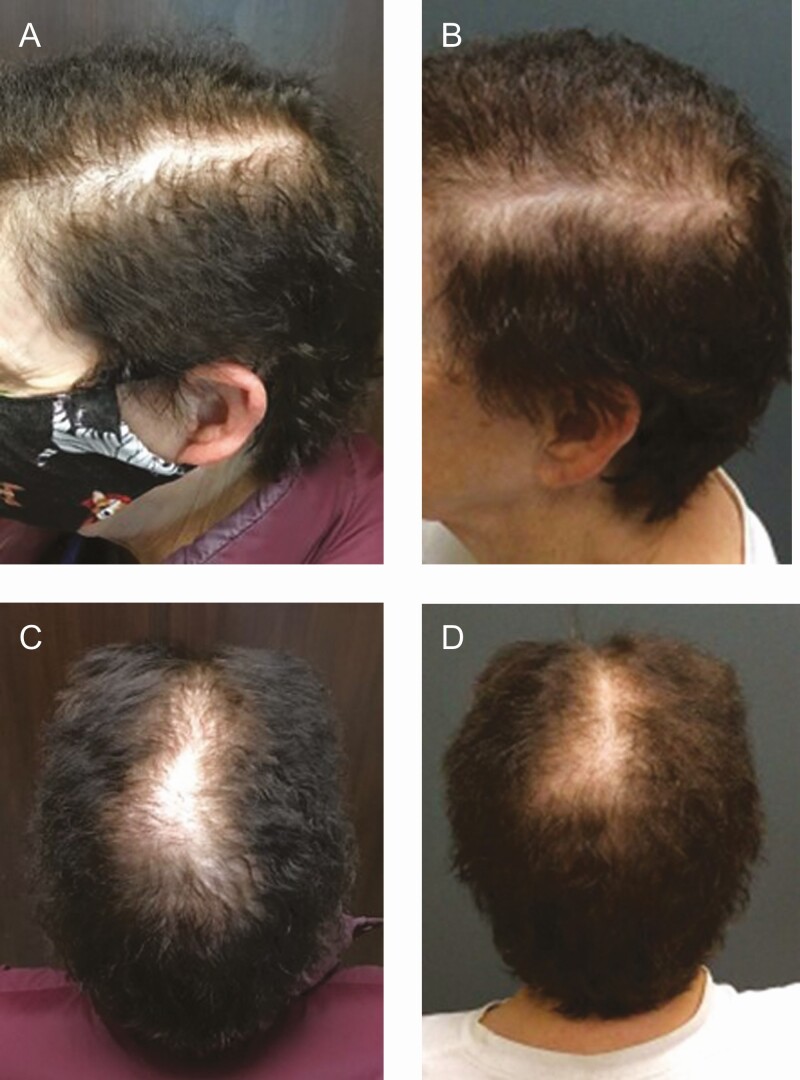
This 72-year-old female developed patterned hair loss (Ludwig II 1) over 3 years and responded initially to finasteride (5 mg/day) and 5 sessions of platelet-rich plasma treatments. (A, C) Hair shedding and loss recurred about a year following her initial responses. (B, D) The patient received 2 mL of XoFlo (1:2 normal saline dilution [Direct Biologics, LLC, Austin, TX]) and observed significant hair growth at 6 months. There were no adverse events.

**Figure 2.**
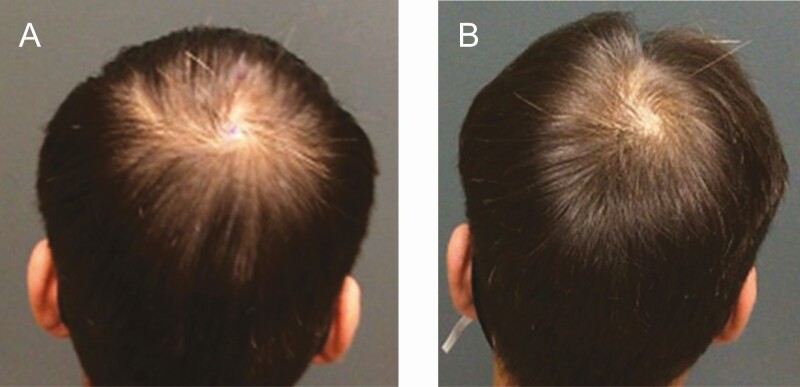
(A) This 27-year-old male developed patterned hair loss (Norwood-Hamilton III) over 7 years and responded to topical 5% minoxidil and dutasteride. Hair shedding and loss resumed 2 to 3 years later. The patient received 5 mL of undiluted Exoflo and responded with significant hair growth after 4 months. (B) The patient is shown 1 year posttreatment. No adverse events were recorded.

The applications of stem cell and non-cell therapies in regenerative aesthetic medicine continue to be confusing for physicians because of ill-defined, misunderstood, and often misleading information in this evolving field of medical practice. On May 31, 2021, the US FDA enacted a policy change that all allogeneic stem cell therapies and their biologic products are regulated under the Federal Drug and Cosmetic Act Section 351 and are not approved for clinical use without an approved IRB, Investigational New Drug Application (IND), and a Biologic License Application (BLA).^52-55^ This retrospective clinical case study was completed before the FDA’s decision under the auspices of a retrospective IRB to investigate this product’s safety and efficacy in hair restoration.

## METHODS

### Study Design

The study considered over 65 male and female candidates in the author’s (G.H.S.) private practice from which subjects were selected who met inclusion (Table 1) and exclusion (Table 2) criteria. Selected subjects were enrolled in this retrospective open-label, nonblinded, nonrandomized, investigator-initiated primary safety and efficacy trial from February 14, 2020, to April 21, 2021. The study protocol was reviewed and approved by the IRB of the Institute of Regenerative and Cellular Medicine (Protocol number: SA-EXO-001, Approval Number: IRCM-2021-292, May 12, 2021). Patients signed informed consents that complied with the standards of the Bill of Rights, in accordance with the Declaration of Helsinki, the Health Insurance Portability and Accountability Act (HIPPA). By signing the provided written consent and release forms, patients also agreed to the use and analysis of their data for presentation and publication. All adverse outcomes were reviewed by the IRB oversight board. Each patient received an initialed copy of their consent forms and protocol. Patients were informed of their financial responsibilities by participation in the study. During the final evaluation period of at least 6 months, each subject’s response was defined as “worse” (progression of hair loss, density, and thinning), “stable” (no change in hair loss, density, or thinning), or “growth” (observed or trichoscan-measured improvement in hair growth, density, and thickness). The basis for grouping clinical responses into these categories was based on the assessment of blinded global photographs, trichoscans, and questionnaires.

**Table 1.** Inclusion List

Characteristics
Males and females, aged 18-80 years and in good health.
Male Pattern Hair Loss (MPHL) Grades III to IV based on Norwood-Hamilton Scale
Female Pattern Hair Loss (FPHL) with early limited or diffuse hair loss consistent with Grades I-3 to III based on the Ludwig Scale
Patients who experienced worsening responses or were in remission on minoxidil, finasteride, dutasteride, and spironolactone after a year on therapy and continued hair loss at least 6 months off medication(s).
Patients on supervised therapies consisting of estrogen/progesterone/testosterone/other pituitary replacement therapy or thyroid replacement therapy and experiencing hair thinning and loss on replacement therapies.
Patients who were treated with follicular unit extraction transplantation and experiencing hair thinning and loss in the transplanted and non-transplanted scalp a year after surgery.
Patients who experienced worsening responses or were in remission after platelet-rich plasma (PRP) treatment(s) after a year on therapy and continued hair loss at least 6 months off PRP treatment.
Patients who were treated with fat grafting, mechanically derived nanofat cells (_t_SVF), or enzymatically derived SVF (_c_SVF) and were experiencing hair loss at least a year after the last treatment.
Absence of physical or psychological conditions unacceptable to investigator.
Patients of childbearing potential who had a negative urine pregnancy test and were willing to use an acceptable method of birth control (barrier methods with spermicidal agent, hormonal methods, intrauterine device, and abstinence during the study).
Willingness and ability to provide written consent for study, photography, and HIPAA authorization before performance of any study-related procedure.

HIPAA, Health Insurance Portability and Accountability Act.

**Table 2.** Exclusion Criteria

Characteristics
Patients who have a diagnosis of telogen effluvium (generalized shedding of hair) or any cicatricial (burn scars) or inflammatory alopecia, or immunogenic-related alopecia (alopecia areata).
Patients who have a sensitive, irritated, or abraded scalp area.
Known allergic reaction to components of study treatment.
History of uncontrolled autoimmune disease, diabetes mellitus, or any cancer.
Use of systemic agents that increase bleeding/clotting or disorders associated with these effects.
Clinically significant medical or psychiatric illness currently as determined by the investigator.
Any disease or condition (medical or surgical) that, in the opinion of the investigator, might compromise hematologic, cardiovascular, pulmonary, renal, gastrointestinal, hepatic, or central nervous system function; or any condition that would place the subject at increased risk.
Pregnant or lactating females; females trying to become pregnant.

### Assessment Criteria

#### Body Mass Index

The total body fat was analyzed by applying the Futrex Light Wand (Hagerstown, MD) at a standardized point on the mid-point of the bicep muscle by a single technician (Margaret Gaston) (Table 3). The near-infrared light beam’s level was computed to reflect the overall body fat level, based on age and gender. The correlation coefficient of percent body fat as predicted by the Futrex near-infrared method was 0.94 (*P* ˂ 0.01). The average of 3 measurements provided the final changes in the percentage of total body fat and BMI values throughout the study.

**Table 3.** Clinical Documentations

Determinants at baseline (V_0_) and month-6 (V_6_)
BMI (kg/m^2^)
Weight measurements (kg)/height measurements (m)
Standardized digital photography
Computerized trichoscans
Subjective questionnaires
IGAIS (Investigator Global Aesthetic Improvement Scale)
PGAIS (Patient Global Aesthetic Improvement Scale)

#### Standardized Photography and Trichoscans

All photographs were taken with a high-quality digital 16 MAGA-PIXELS high-resolution camera and color macro-lens that provided 12× and 36× magnification for macro-hair images at standardized angles, positions, and distances in the same room against a solid backdrop with tangential lighting to highlight features (External Medical Camera, CAPILY Institute, Los Angeles, CA). All photographs were compiled by removal of identifying information before their assessment. Each of 3 blinded evaluators was instructed to judge unlabeled pretreatment and posttreatment photographs of each patient’s scalp for changes in hair density and growth patterns. The evaluation in each scalp was scored as “improvement (mild, modest),” “no change,” or a “worsening” of hair density and growth coverage.

Trichoscans were performed in all subjects by a trained technician with the use of Machine learning, computer vision algorithms for hair image processing, and the use of software application to automatically render diagnostic measurements of hair density, follicle diameter, and terminal/vellus hairs within a 0.8-cm^2^ located at the frontal hairline, mid-scalp, and crown with the same External Medical Camera System. All hairs with a diameter of >40 µm were categorized as terminal hair, and all hairs with lesser diameter were categorized as vellus hairs. An independent technician outside the office performed the calculated measurements from each site which were averaged to provide final numbers that compared the effects between baseline (V_0_) and month 6 (V_6_).

#### Satisfaction Questionnaires

Secondary endpoints included investigator and patient assessments of global and hemi-scalp changes in hair density and thickness at baseline and follow-up visits. For each patient, paper surveys were identified by a unique number and evaluation date and distributed as a batch before the starting date. Each patient was asked to fill out their surveys in the privacy of their homes before their evaluation sessions. The investigator filled out his assessment for each patient in privacy after each evaluation session. The registered nurse collected all subject and investigator surveys which were calculated at the end of the study by the statistician utilizing the designated identification number for each patient on a 6-point satisfaction scale for the entire scalp (Table 4).

**Table 4.** Investigator or Patient Global Aesthetic Improvement Scales (IGAIS or PGAIS)

Scale	Level	Description
1	Extremely dissatisfied	Marked worsening in appearance from the initial condition
2	Very dissatisfied	Appearance worse than the original condition
3	Dissatisfied	Appearance is essentially the same as the original condition
4	Satisfied	Obvious improvement in appearance from the initial condition
5	Very satisfied	Marked improvement from initial appearance
6	Extremely satisfied	Optimal cosmetic result for the procedure in this subject

### Extracellular Vesicles Product

XoFlo (Direct Biologics, LLC, Austin, TX) is an aseptic human bone marrow mesenchymal stem cell-derived EV isolate. The donated human bone marrow tissue allograft is a minimally manipulated single-donor-per-lot tissue product and contains no animal products, chemicals, or drugs, and is manufactured under strict cGMP guidelines for the absence of communicable diseases in the processing and isolation of the product. XoFlo is marked solely for homologous use and packaged as a frozen solution in 1-, 2-, and 5-mL vials. ExoFlo, the same product designated as XoFlo in this study, is labeled for only COVID clinical trials that are currently being conducted and FDA-approved under Investigational New Drug (IND) #: 21669 by its manufacturer, Direct Biologics.

### Surgical Technique

Surgery was performed under local anesthesia in an office operating suite. Preoperative dots with a marking pen outlined the regional distribution of hair loss or thinning, such as in the frontal-temporal zones, mid-scalp, and crown areas. An aseptic preparation was performed with the application of isopropyl alcohol solution (70%). An anesthetic ring block with 0.5% lidocaine-containing epinephrine (1:200,000), buffered with 8.4% sodium bicarbonate solution (8:2 ratio), was infiltrated in the subdermal plane using a 30-gauge needle attached to a tuberculin syringe. The average total volume injected varied between 5 to 7 mL. With this technique, the surgical field was maintained with less bleeding from subsequent microneedling. The content in the frozen vial was thawed at room temperature within 10 minutes and withdrawn into a larger syringe in which bacteriostatic 0.9% normal saline was added to the original XoFlo volume as needed in ratios ranging from 1:2 up to 1:10. This diluted volume allowed sufficient treatment of XoFlo from smaller to larger areas of the scalp. The author preferred to use undiluted 2 or 5 mL volumes so that the highest number of EVs was localized to treat alopecic areas.

The XoFlo preparation was loaded into separate 1-mL tuberculin syringes to which was attached a 32-gauge short needle. Approximately 0.08 to 0.1 mL was deposited in the intradermal plane at each dot from which an estimated spread of solution up to 2.5 cm of the solution was anticipated. Light scalp massaging further distributed the EV solution more evenly throughout the scalp. Patients, who consented to 2.5-mm microneedling (SkinPen, Crown Aesthetics, Dallas, TX) over the entire treated scalp, received 2 to 3 passes with the stamping technique to produce visible blood staining at the openings. The scalp was washed with warm sterile water and towel-dried. Patients, who consented to a 678-nm Low-Level Light-Therapy (LLLT) light treatment from an FDA-cleared laser diode helmet device (TheraDome LH80, Pleasanton, CA), were immediately treated for a single 20-minute session. Patients were recommended to continue their previous scalp medications or topicals to reduce any anticipated withdrawal shock of their hair into quiescent phases.

## RESULTS

### Baseline Patient Characteristics

Baseline demographics and clinical characteristics are reported in Table 5. Nine males (average age: 43.3 years; range, 27-76 years) and 22 females (average age: 62.9 years; range, 28-87 years) were treated from November 3, 2020, to April 21, 2021. None of the 31 patients were excluded for protocol infractions or COVID-19 infections. Patients maintained their weights within 1 to 5 pounds throughout the 6-month study from their baseline BMIs: males (average BMI: 25.1 kg/m^2^; range, 19.6-28.9) and females (average BMI: 22.7 kg/m^2^; range, 18.6-35.1). Percentages of patients by race were 71% Caucasian, 20% Asian, 6% Hispanic, and 3% Middle-Eastern. Years of alopecia experienced in males (average 9.4 years; range, 3-20 years) and females (average 7.2 years; range, 1-25 years) were estimated based on recollection. The Norwood-Hamilton classification for MPHL ranged from III (6 patients) and IV (3 patients), while Ludwig classification for FPHL ranged from I-3 (1 patient), II-1 (13 patients), II-2 (5 patients), and III (3 patients).

**Table 5.** Clinical Demographics

Demographics	Female	Male
Number	22	9
Average age (range, years)	62.9 (28-80)	43.3 (27-7)
Average BMI (range, kg/m^2^)	22.7 (18.6-35.1)	25.1 (19.6-28.9)
Ethnicity		
Middle-Eastern	0	1
Hispanic	2	0
Asian	5	1
Caucasian	15	7
Average years alopecia (range)	7.2 (1-25)	9.4 (3-20)
Classification		
Norwood-Hamilton	n/a	III (6) IV (3)
Ludwig	I-3 (1) II-1 (13) II-2 (5) III (3)	n/a

Baseline characteristics of male and female subjects treated over a 13-month period in an open-labeled, nonblinded, nonrandomized, retroactive IRB-approved safety and efficacy trial.

### Age Influence on XoFlo Response

Although 20 female patients between 20 and 80 years of age demonstrated either growth (12/22 patients) or stable maintenance (8/22 patients) of hair density after a single XoFlo treatment, the greatest number clustered around 50 to 70 years of age (Table 6). Two female patients (38 years old; 59 years old) reported progressive hair loss after treatment (see next section below). Although 9 male patients between 20 and 70 years demonstrated either growth (8/9 patients) or stable maintenance of hair loss (1/9 patients) after a single XoFlo, most responders were between 20 and 50 years of age.

**Table 6.** Age Influence on XoFlo (Direct Biologics, LLC, Austin, TX) Responses

No. and sex of patients	Age (years)	Worse (no.)	Stable (no.)	Growth (no.)
22 females	20	—	—	1 (++)
	30	1	—	—
	40	—	—	—
	50	1	1	3 (++)
	60	—	3	6 (+)
	70	—	3	2 (+)
	80	—	1	—
	%	9.0%	36.4%	54.5%
9 males	20	—	—	1+++
	30	—	—	2++
	40	—	—	2++
	50	—	—	1++
	60	—	—	—
	70	—	—	2+
	80	—	—	—
	%	0%	11.1%	88.9%

### Potential Factors for ExoFlo Failures

Two female patients demonstrated progressive loss of hair density and growth after receiving a variety of FDA-approved and other drugs for hair loss. The 38-year-old patient with over 10 years of alopecia responded neither to combination therapy of 4 drugs over 5 years nor later to 4 sessions of platelet-rich plasma treatments while on spironolactone and dutasteride medications (Table 7). Each patient was asked to discontinue any hair loss treatments a year before initiating a single XoFlo treatment. Both patients were observed to be poor responders to XoFlo therapy from serial protocol evaluations and trichoscan measurements every 3 months for a year. Patients continued treatments for severe hormonal imbalances and major stress disorders during all hair stimulation therapies.

**Table 7.** Potential Factors for XoFlo (Direct Biologics, LLC, Austin, TX) Failures

Patient age (years)	Alopecia years	Ludwig	Influence of medications and disease factors to XoFlo effects	Influence of surgical factors to XoFlo effects	ExoFlo volume dilution	Microneedling
Patient (38)	10 years	III	• Severe hormonal imbalance • Failure to minoxidil, finasteride, spironolactone, and dutasteride • Severe stress	Failure to 4 platelet-rich plasma (PRP) sessions	2 mL volume 1:2 mL dilution	None
Patient referred (59)	1 year	III	• Severe hormonal imbalance • Failure to minoxidil, finasteride, steroid, and methotrexate • Severe stress	None	5 mL no dilution	1 session

### Influence of Duration of Alopecia on XoFlo Response

Thirteen female patients with less than 7-year history of alopecia demonstrated either growth (9/22) or stable maintenance (4/22) of their hair density after a single XoFlo treatment Table 8. Moreover, a lesser number of female patients with over 10 years of hair loss were still capable of either hair growth (3/22) or maintenance (4/22) after treatment. In 2 patients, the wide differences in durations of alopecia (1 and 10 years) did not appear to play a significant role in predicting outcomes because other adverse factors may have contributed to the “worse” results after XoFlo treatment. In MPHL, the impact that duration of alopecia plays may be clearer than that observed in FPHL. In this study, 7 of 9 male patients with less than 8-year history of alopecia and a briefer time for hair loss exhibited increased hair growth and density after treatment. However, 2 male patients with over a 20-year history of hair loss also noticed stabile maintenance of their alopecia but not growth after treatment.

**Table 8.** Potential Influence of Long Durations of Alopecia on Responses to XoFlo (Direct Biologics, LLC, Austin, TX) Treatment

No. and sex of patients	Alopecia (years)	Worse (No.)	Stable (No.)	Growth (No.)
22 females	1	1		
	2			1
	3			3
	4			
	5		2	4
	6		1	
	7		1	1
	8			
	9			
	10+	1	4	3
	20+			
	%	9.0%	36.4%	54.5%
9 males	1			
	2			
	3			2
	4			
	5			3
	6			1
	7			
	8			1
	9			
	10			
	20		2	
	%		22.2%	77.8%

### Influence on Stage of Alopecia on XoFlo Response

The clinical staging of alopecia may not necessarily correspond with the duration of alopecia because of the unpredictable rate and proportion of hair loss that are often observed from early hair thinning to recognizable patterns of obvious hair loss in male and female pattern alopecia Table 9. Despite these characteristic occurrences, 9 of 22 female patients with early Ludwig stages of hair loss (I-3, II-1) and only 3 of 22 patients with more advanced stage-II-2 pattern alopecia observed hair growth and increased density after treatment, while 8 other patients with Ludwig II-1 to extensive diffuse Ludwig stage III of hair loss were found to have stable maintenance of hair loss after one treatment. As previously mentioned, 2 female patients with extensive Ludwig III stages of hair loss and associated adverse medical factors experienced “worse” results after XoFlo treatment. On the other hand, 8 of 9 male patients with early stages of Norwood-Hamilton pattern hair loss (III-IV) exhibited increased hair growth and density, while the remaining patients (Norwood-Hamilton III) experienced stable maintenance of hair loss after a single treatment. Although the data suggest that there may be a potential advantage in treating earlier rather than advanced stages of MPHL or FPHL, further multicentered studies with a larger number of patients, wider range of alopecia, and longer follow-ups will be required to substantiate these preliminary findings.

**Table 9.** Influence on Stage of Alopecia on XoFlo (Direct Biologics, LLC, Austin, TX) Response

Sex of patients	Scale	Level	Worse (no)	Stable (no)	Growth (no)
Females	Ludwig	I-3	—	—	1
		II-1	—	5	8
		II-2	—	2	3
		III	2	1	—
		%	9.1%	36.4%	54.5%
Males	Norwood-Hamilton	III	—	1	5
		V	—		3
		%	—	11.1%	88.9%

### Influence of XoFlo Volume (mL) on Response

A greater hair growth response can be anticipated when a greater number of paracrine factors are made available after the administration of larger volumes of EVs (Table 10). Although 12 of 22 females exhibited increased hair growth and density, the fraction that received 5-mL ExoFlo observed a larger amount of hair growth, follicle diameter, and density than those treated between 2 and 4 mL of Exoflo. Furthermore, 8 of 22 patients observed stabilization of their hair loss after receiving either 2, 3, or 5 mL of ExoFlo. It is speculated that the 2 female patients who were worse after treatment had other unfavorable medical factors that possibly worked against a positive paracrine outcome. Six of 9 male patients, who were treated with volumes 5, 7, and 8 mL, responded with more hair growth than that observed in 2 other patients treated with 1 to 2 mL of XoFlo. One male, who received 2 mL of XoFlo, demonstrated less shedding and more stabilization of hair loss. The preliminary data suggest again that the use of larger rather than smaller volumes of ExoFlo resulted in more predictable and robust outcomes of hair density in MPHL and FPHL.

**Table 10.** Potential Influence of Concentration of Extracellular Vesicles/Undiluted Volumes of XoFlo (Direct Biologics, LLC, Austin, TX) on Responses to Treatment

Sex	ExoFlo (Direct Biologics, LLC, Austin, TX) volume	Worse (no)	Stable (no)	Growth (no)
Female	1 mL			
	2 mL	1	6	7 (+)
	3 mL		1	
	4 mL			1 (++)
	5 mL	1	1	4 (+++)
	6 mL			
	7 mL			
	8 mL			
	%	9.0%	36.4%	54.5%
Male	1 mL			1 (+)
	2 mL		1	1 (++)
	3 mL			
	4 mL			
	5 mL			4 (+++)
	6 mL			
	7 mL			1 (+++)
	8 mL			1 (+++)
	%	0%	22.2%	77.8%

### Influence of Dilution of XoFlo on Response

Although the total amount of paracrine factors remains the same regardless of its dilution with normal saline, the delivery of a greater concentration of exosomes in a full-strength volume is anticipated to produce a superior response in a fixed area of alopecic scalp (Table 11). In this manner, 11 of 22 female patients observed greater hair growth and density with either undiluted or a 1:2 diluted solution, while 1 patient responded with lesser growth after a 1:4 dilution treatment. Moreover, stable maintenance of hair loss was reported in 8 other patients who received undiluted XoFlo or dilutions ranging from 1:2 to 1:5. The abovementioned 2 female patients, who experienced worsening outcomes, received either 1:2 dilution or undiluted 5 mL of Xoflo. Eight male patients who received either undiluted (6/9) or 1:2 dilution (2/9) responded with a robust increase in hair density and diameter, while only 1 patient reported stable maintenance of hair loss after receiving a 1:2 dilution of XoFlo. Once again, the application of pure or minimally diluted solutions rather than higher mixtures predictably yielded greater hair growth and density in MPHL and FPHL patients.

**Table 11.** Influence of Dilution of XoFlo (Direct Biologics, LLC, Austin, TX) on Response

No. and sex of patients	Influence of dilution ratios to XoFlo Effect	Worse	Stable	Growth
22 females	None	1	2	5
	1:2	1	2	6
	1:3	—	2	—
	1:4	—	—	1
	1:5		2	—
9 males	None	—	-	6
	1:2	—	1	2
	1:3	—	—	—
	1:4	—	—	—
	1:5	—	—	—

### Influence of Previous Drug and Disease Factors in Females on XoFlo Response

It is yet to be proven if susceptible hairs that responded to the usage of FDA-approved hair medications or to medical therapies for systemic diseases would also respond in turn to paracrine-stimulating factors in ExoFlo (Table 12). That said, 7 female patients who experienced hair growth after topical minoxidil, finasteride, dutasteride, and spironolactone treatments and were in remission for a year also responded to XoFlo, while 1 patient, who did not achieve any significant changes in hair density after minoxidil, noted less hair loss after XoFlo treatment. The above-mentioned 2 female patients, who experienced worsening effects after XoFlo, were reportedly unresponsive to hair stimulation drugs and were also resistant to the usage of intradermal steroids and oral methotrexate. In addition, both remained under medical care for severe stress and hormonal imbalances. Of interest, 4 females who were on effective therapies for medical diseases (Hashimoto’s disease, exophthalmos from hypothyroidism, diabetes mellitus, and hormonal imbalance) demonstrated at least stable maintenance of their hair loss after XoFlo treatment. One patient with diet-controlled diabetes mellitus responded to treatment.

**Table 12.** Influence of Previous Drug and Disease factors in Females on XoFlo (Direct Biologics, LLC, Austin, TX) Responses

Influence of drug factors to XoFlo effect	Worse	Stable	Growth
Minoxidil (positive response)	—	—	4
Minoxidil (minimal to no response)	2	1	—
Finasteride (positive response)	—	—	1
Finasteride (minimal to no response)	2	—	—
Dutasteride (positive response)	—	—	1
Dutasteride (minimal to no response)	1	—	—
Spironolactone (positive response)	—	—	1
Spironolactone (minimal to no response)	1	—	—
Steroid/Methotrexate	1	—	—
Hashimoto’s disease	—	1	—
Hypothyroidism (Exophthalmos)	—	1	—
Diabetes Mellitus	—	1	1
Hormonal Imbalance (severe HRT)	2	1	—
Major stress	2	—	—

HRT, hormone replacement therapy.

### Influence of Previous Drug and Disease Factors in Males on XoFlo Response

Seven male patients who experienced hair growth with topical minoxidil or oral finasteride and dutasteride and were in remission for a year also responded to treatment (Table 13). One patient with controlled juvenile diabetes, who responded to the previous usage of minoxidil and then was observed to be in remission, exhibited hair growth after XoFlo treatment.

**Table 13.** Potential Responses in Males to Previous Medications for Hair and Presence of Diseases Known to Affect Hair on Responses to XoFlo (Direct Biologics, LLC, Austin, TX) Treatment

Drug factors	Worse	Stable	Growth
Minoxidil (positive response)	—	—	5
Minoxidil (minimal to no response)	—	—	—
Finasteride (positive response)	—	—	3
Finasteride (minimal to no response)	—	—	—
Dutasteride (positive response)	—	—	3
Dutasteride (minimal to no response)	—	—	—
Disease factor	—	—	—
Diabetes mellitus	—	—	1

### Influence of Previous Surgical Procedures on XoFlo Response in Females

It would be anticipated that patients who responded to paracrine factors in PRP would probably benefit from similar growth factors and cytokines in XoFlo (Tables 14, 15). That said, 5 female patients, who demonstrated hair growth after 2 to 6 sessions of PRP and were in remission for a year, also exhibited increased hair density after ExoFlo treatments, while 2 patients who showed no response to PRP treatments reported less shedding after ExoFlo treatment. The previously mentioned female patient, who reported an increased loss of hair after 4 PRP sessions, also observed increased shedding after XoFlo treatment. Another patient, who responded with increased hair density after 1 session of fat grafting and PRP to her scalp 3 years before and thereafter exhibited hair loss, also noticed increased hair density after XoFlo treatment. Four of 9 male patients who benefitted from 3 to 6 sessions of PRP but were in remission for a year demonstrated increased hair density after a single XoFlo treatment. Of the 2 male patients who had undergone previous follicular unit extraction (FUE) hair transplantations, and increased hair growth after fat grafting and PRP treatments at least 3 years before and in remission, 1 reported increased hair density after XoFlo treatment, while the other male exhibited less hair loss after XoFlo treatment. 

**Table 14.** Influence of Surgical Factors on XoFlo (Direct Biologics, LLC, Austin, TX) Effects in Females

Surgical factors	Worse	Stable	Growth
Platelet-rich plasma			
1 session	—	1	—
2 sessions	—	—	1
3 sessions	—	1	1
4 sessions	1	—	—
5 sessions	—	—	2

**Table 15.** Influence of Surgical Factors on XoFlo (Direct Biologics, LLC, Austin, TX) Effects on Males.

Influence of surgical factors to XoFlo effect	Worse	Stable	Growth
Platelet-rich plasma (PRP)			
1 session			
2 sessions			
3 sessions			2
4 sessions			1
5 sessions			
6 sessions			1
Hair transplantation + fat grafting + PRP		1	1

### Influence of Microneedling on XoFlo Response

After XoFlo treatments, the beneficial influence of microneedling in female and male patients was inconclusive (Tables 16, 17).

**Table 16.** Influence of Microneedling to XoFlo (Direct Biologics, LLC, Austin, TX) Effect in Females

Influence of microneedling (2.5 mm)	Worse	Stable	Growth
MN+	1	5	8
MN−	1	3	4

MN, microneedling.

**Table 17.** Influence of Microneedling to XoFlo (Direct Biologics, LLC, Austin, TX) Effect in Males

Influence of microneedling (2.5 mm)	Worse	Stable	Growth
MN+	—	1	3
MN−	—	—	5

MN, microneedling.

### Trichoscan Measurements

At 6 months, trichoscans were performed in 7 female and 4 male patients who responded to treatment. The percent changes from baseline in hair density, terminal hair density, vellus hair density, and follicle diameter were analyzed at the frontal-temporal scalp, mid-scalp, vertex, and control occiput. Increases in percent change in hair densities (11.1%-24.2%), terminal hair densities (16.4%-45.5%), vellus hair densities (18.4%-36.4%), and follicle diameter (9.4%-32.3%) were most consistently measured in the frontal-temporal scalp and least in the occipital scalp.

Trichoscans at 6 months were also done in 3 female and 1 male patient, who observed no significant change or experienced hair loss after treatment (data not shown). The percent changes from baseline in hair density (−1.6% to +5.3%), terminal hair density (−3.4% to +3.3%), vellus hair density (−5.1% to +1.7%), and follicle diameter (−5.3% to +4.6%) were consistently below percent changes in the occipital region (8.7% to 15.8%). The quantitative data from these 15 patients need confirmatory measurements from a larger cohort to validate these preliminary observations.

### Global Questionnaire Assessments

Patients and investigator completed the satisfaction questionnaires at baseline and month 6 on a 6-point scale (Table 18). Each subject’s Patient Global Aesthetic Improvement Scale (PGAIS) value closely aligned with Investigator Global Aesthetic Improvement Scale (IGAIS) determination at both evaluation periods. Of the 22 female patients, 2 remained “very dissatisfied,” 8 observed “satisfied,” and 12 were “very satisfied” with their results. Of the 9 male patients, 1 remained “dissatisfied,” 5 were “satisfied,” and 3 were “extremely satisfied” with their treatments.

**Table 18.** Global Questionnaire Assessments

Sex of patients	No. of patients	IGAIS/PGAIS baseline	IGAIS/PGAIS month 6
Male	1	3	3
	5	3	4
	3	3	5
Female	2	1	2
	8	3	4
	12	3	5

IGAIS, Investigator Global Aesthetic Improvement Scale; PGAIS, Patient Global Aesthetic Improvement Scale.

### Tolerability and Safety

Patients did not experience significant adverse events such as fever, myalgias, chills, and fatigue. Transient swelling and localized pain to the scalp for a day were most likely from microneedling at a depth to produce bleeding at the entry points. No scalp cellulitis occurred after the procedure. Patients were not treated prophylactically with oral antibiotics or pain medications. They were advised to shower the day after the procedure with their usual shampoo. Patients returned to their normal physical activities within 24 hours.

## DISCUSSION

Stem cells and their products have shown promise for hair restoration based on their paracrine signaling in regulating hair cycling, reactivation of quiescent miniaturized follicles, de novo formation of new follicles, and reversing the course of apoptotic follicles.^56^ Although the precise bioactive molecules that synchronize discrete stem cell populations within the follicle to be active, quiescent, stimulatory, or inhibitive are unknown,^57-62^ they primarily consist of pivotal growth factors, cytokines, messenger RNAs, and microRNAs secreted by specialized follicular stem cells,^63-66^ extrinsic mesenchymal,^21,25,67^ and hematogenous stem cell sources.^24^ The earliest strategy of using ADSCs,^68-70^ bone marrow-derived regenerative cells,^71^ or PRPs^72-75^ to restore hair growth may not be the cells themselves but their secreted factors.^19^ In fact, recent investigations have begun to focus on the clinical applications of secretomes, as a cell-free alternative to stem cell-based approaches because they possess significant advantages over cell-based therapies.^76,77^ The second strategy employed the use of secretomes, obtained from conditioned media of cultured ADSCs, which not only provided salutary paracrine effects on neighboring wound healing cells by their angiogenic, hematopoietic, anti-apoptotic, fibroblastic, and pro-inflammatory properties^78,79^ but also directly impacted follicular growth.^34,35,80-82^

In the past 3 decades, the third approach by which stem cells act in a paracrine fashion has emerged that involves the intercellular transfer of EVs, one of which has been identified as exosomes. Recent articles^83,84^ have described the status of isolation, characterization, molecular composition, and biogenesis of these lipid-lined sacs that contain a diverse number of growth factors, cytokines, micro-RNA, and messenger RNA. These bioactive molecules are known to modulate cell-cell communications for homeostasis, immune signaling, angiogenesis, inflammation, senescence, proliferation, and differentiation.^50^ As expected, EV effects in vitro and in animal studies were first observed in stem cell-conditioned media from deer antler mesenchymal stem cells,^85^ mouse-bone marrow mesenchymal cells,^86^ and ADSCs^87^ that increased *Wnt3a* mRNA expression in dermal papilla (DP) cells. Further investigations demonstrated that EVs isolated from DP cells increased proliferation and differentiation of cells in the DP,^88^ matrix,^89^ outer root sheath cells,^90^ shaft elongation,^90^ and prolongation of the anagen phase.^89^ Although these preclinical investigations strongly suggested that EVs could promote hair follicle growth and development, there are currently no published clinical IRB-approved trials reporting on their safety and efficacy in humans except for preliminary presentations^91,92^ and a review article.^47^

This retrospective open-label trial gathered additional information on the safety and efficacy of XoFlo treatments on 22 females and 9 males under a retrospective-approved IRB. The author accepted patients who complained of progressive hair loss and thinning or were disappointed by the results of previous medications or surgical procedures. To evaluate the safety of this new therapy, patients were cleared by their physicians of any health risks and were followed for any side effects or significant adverse reactions after previous medical treatments and to XoFlo injections. To evaluate the efficacy, the use of global photographs, trichoscans, and follow-up questionnaires were used to interpret results that may be influenced by the patients’ different ethnic backgrounds, ages, stage of classifications, durations of alopecia, responses to previous treatments or impact of coexisting disease factors, the amount of delivered XoFlo, and finally the effects of wound healing from microneedling. Because of the small sampling numbers and short evaluations between 6 months to a year, however, provisional conclusions were speculatively defined as “worse,” “stable,” or “growth” trends from baseline values.

A binary treatment response of reduced shedding or increased hair growth was observed in females over the age of 50 years and in males under the age of 50 years. The reasons for this age dichotomy were unclear. Several studies^93,94^ have suggested that males significantly responded to PRP treatments with a lesser effect than females. This gender-specific effect might be due to significant multifactorial conditions observed in especially younger females related to hair loss, which included oral contraceptive medications, premenopausal status, postpartum hormonal imbalances, dietary changes, thyroid and pituitary abnormalities, and environmental stresses. The higher number of growth responses to XoFlo in both female and male patients occurred with shorter histories of alopecia ˂8 years or earlier Norwood-Hamilton/Ludwig stages of hair loss. This observation may be anticipated because of the greater amount of irretrievable hair loss that needed to be reversed in the advanced stages of alopecia. Furthermore, the use of larger, as well as undiluted volumes of XoFlo, tended to result in growth or stable maintenance of hair loss in higher numbers of male or female patients. This outcome might be due to the provision of greater numbers of EVs at each injection site. The growth responses to approved medications for hair growth, such as minoxidil or finasteride, might serve to be indicators for a similar response to exosomes as both have been shown to increase the activity of stem cell activities within the dermal papilla and bulge domains. Similarly, patients who exhibited hair growth after a series of PRP sessions would be more likely to respond to exosome treatments since similar biomodulators were involved in their paracrine effects. The presence of entities known to have a negative impact on hair growth such as autoimmune diseases, endocrine imbalances and diseases, and severe stress disorders was speculated to contribute to poorer outcomes after EV treatments and deserve to be further studied. Lastly, the effects of microneedling at a 2.5-mm needle depth resulted in mixed and inconclusive results in both female and male patients. The effects of microneedling in their wound-healing capacity might be insufficient to augment the beneficial effects of EVs. Most patients observed hair growth between the third to sixth months and did not require a booster session after a year. This study did not specifically address whether hair growth and increased density resulted from prolonging the anagen phase, shortening the telogen quiescent phase, facilitating transitions from vellus hairs, rescuing dormant exogen hair, or stimulating the formation of de novo hair follicles.

Trichoscan measurements were obtained in 15 of the 31 patients. Results showed that average hair densities, terminal hair densities, vellus hair densities, and follicle diameters at the 6-month follow-up period were greater than that observed in the control occipital area in patients that clinically responded to treatment. On the other hand, 4 patients that were found to have minimal to no response to treatment also had lower average percent changes in comparison to the average percent changes in the control occipital site. The remaining 16 patients in the study were unable to have their final trichoscan evaluations because of the pandemic shutdown.

Most patients, including the investigator, assessed their results as “satisfied” to “very satisfied” after a single treatment. Longer follow-ups may determine whether a booster is required to increase and sustain their hair density over time.

## CONCLUSIONS

This retrospective IRB-approved exosome study demonstrated preliminary promising and safe results on the treatment of female and male hair loss. The importance of demographic factors, medical conditions, responses to previous hair treatments, and methodologies in protocol design has yet to be determined as indicators to predict responses from therapy. Further multi-center IRB trials with larger numbers, standardized protocols, and longer follow-ups are needed to assess the safety and efficacy of this new therapy. Although the application of cell-free therapy over cell therapy has been discussed, there exist major hurdles that include the need for more evidence-based data to allow phased studies under FDA oversight and application for an Investigational New Drug status. Currently, all stem cell therapies and the use of their biologic paracrine products are not FDA approved and are under strict regulatory oversight.
